# Novel Aerial Manipulator for Accurate and Robust Industrial NDT Contact Inspection: A New Tool for the Oil and Gas Inspection Industry

**DOI:** 10.3390/s19061305

**Published:** 2019-03-15

**Authors:** Miguel Ángel Trujillo, José Ramiro Martínez-de Dios, Carlos Martín, Antidio Viguria, Aníbal Ollero

**Affiliations:** 1 CATEC (Advanced Center for Aerospace Technologies), C/ Wilbur y Orville Wright 19, 41309 La Rinconada, Seville, Spain; aviguria@catec.aero; 2 School of Engineering, University of Seville, Avda. Camino de los Descubrimientos, 41092 Seville, Spain; jdedios@us.es (J.R.M.-d.D.); aollero@us.es (A.O.); 3 La Línea Vertical, Camino de la Ermita 10, 11300 La Línea de la Concepción, Cádiz, Spain; cmartin@lalineavertical.com

**Keywords:** aerial, robot, manipulator, contact, inspection, NDT, ultrasonic, oil and gas, UAV

## Abstract

There is a strong demand in the oil and gas industry to develop alternatives to manual inspection. This paper presents AeroX, a novel aerial robotic manipulator that provides physical contact inspection with unprecedented capabilities. AeroX has a semi-autonomous operation, which provides interesting advantages in contact inspection. In the free-flight mode, the pilot guides the robot until performing contact with its end-effector on the surface to be inspected. During contact, AeroX is in its fully-autonomous global navigation satellite system (GNSS)-free contact–flight mode, in which the robot keeps its relative position w.r.t. the surface contact point using only its internal sensors. During autonomous flight, the inspector can move—with uninterrupted contact—the end-effector on the surface for accurately selecting the points where to perform A-scan measurements or continuous B-scan or C-scan inspections. AeroX adopts an eight-tilted rotor configuration and a simple and efficient design, which provides high stability, maneuverability, and robustness to rotor failure. It can perform contact inspection on surfaces at any orientation, including vertical, inclined, horizontal-top or horizontal-bottom, and its operation can be easily integrated into current maintenance operations in many industries. It has been extensively validated in outdoor experiments including a refinery and has been awarded the EU Innovation Radar Prize 2017.

## 1. Introduction

The oil and gas industry has a large variety of procedures to ensure the safety of its facilities and personnel, and at the same time tightly controlcosts. These procedures involve extensive inspections, many of which should be performed at height and using sensors that require to be in contact with the surfaces being inspected. Contact inspection is traditionally performed by technicians accessing to the specific inspection points using man-lifts, cranes or scaffolds, or rope-access techniques. Plant contact inspections involve very high costs for the oil and gas sector. Even a small refinery requires inspecting several thousands of points each year and at least 30% of them are in areas of very difficult access.

There is a strong demand in the oil and gas industry to develop alternatives to manual inspection. Sensors deployed at selected locations in the plant, such as in [1,2], provide punctual measurements at a high rate, which is suitable for intensive monitoring of few or small critical components but is not ideal for covering/inspecting a full plant. Crawler robots with magnetic wheels have been developed to move on the outer or inner surfaces of pipes or structures [3]. But they are valid only for ferromagnetic surfaces and often have low accessibility for locations at height or isolated from nearby structures.

The aerial robotics solutions for the oil and gas industrial inspections at height have been analyzed in the H2020 EU project AEROARMS (https://aeroarms-project.eu/). Aerial vehicles equipped with robotic arms, which have been called aerial manipulation systems, have demonstrated their capability to grasp and deploy objects, turn valves or assemble structures [4,5,6]. One of the main research challenges in aerial robotics manipulation is to keep stability during physical contact [7,8]. The aforementioned aerial manipulation robots are very promising but are still not ready or robust for their everyday use in industrial conditions.

This paper presents AeroX, a novel aerial robotic manipulator solution for physical contact inspection. It is composed of a robotic vehicle, a six degree-of-freedom (DoF) robotic arm, and a robotic end-effector equipped with wheels and inspection sensors. AeroX provides unprecedented inspection capabilities. Firstly, it can perform contact inspection on surfaces with any position and orientation, including vertical, inclined, horizontal-top or horizontal-bottom. Secondly, the AeroX controller is able to efficiently compensate perturbations thanks to its design, which transmits the surface contact forces and perturbations to the robot center of mass and allows small movements of the aerial part of the robot in every DoF to absorb other perturbations such as wind. AeroX adopts an eight-tilted rotor configuration, which provides high maneuverability, agility, and robustness to rotor failure. Finally but not least, in contrast to existing systems, AeroX has a semi-autonomous operation. During free-flight, the pilot guides the robot and, after contacting the surface with its end-effector, switches to fully-autonomous global navigation satellite system (GNSS)-free contact flight. During contact flight, AeroX flies keeping its position steady w.r.t. the surface contact point with uninterrupted contact, so that when the inspection operator moves the wheels of the end-effector, the aerial robot follows that motions. As a result, AeroX can be easily operated by personnel with low training in robotics and integrated into current maintenance operations in many industries.

**Figure 1:**
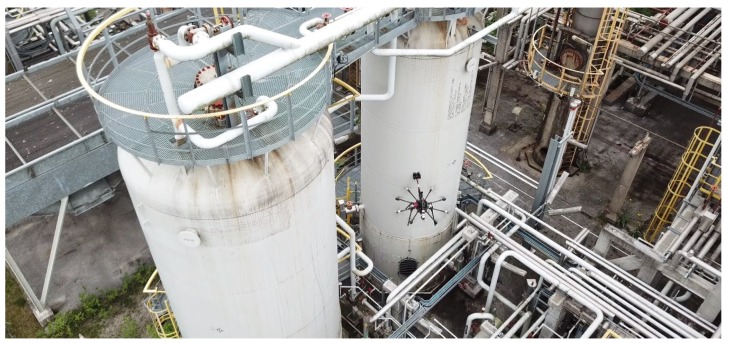
AeroX performing an ultrasonic thickness measurement in a refinery located in Germany in 18 July. The exact location cannot be disclosed due to confidentiality issues.

This paper analyzes the inspection requirements for its use in the oil and gas sector, presents the solution and evaluates its robustness and performance in experiments performed in realistic outdoor scenarios and in a refinery in Germany, see Figure 1. A video of these experiments can be seen at https://youtu.be/Hy45WQ3GLcI. The proposed aerial manipulator has been patented in [9] and has recently been awarded the EU Innovation Radar Prize 2017 (https://www.innoradar.eu/innoradarprize).

This paper is structured as follows. Section 2 summarizes the state of the art of technological solutions for non-destructive testing (NDT) inspection in the oil and gas sector focusing on aerial manipulators. The proposed aerial manipulator solution is described in Section 3 and Section 4. The operation during a typical inspection process is summarized in Section 5. The experimental validation and robustness analysis are presented in Section 6. Section 7 summarizes the conclusions and future work.

## 2. Related Work

Periodic inspections are required to ensure suitable and safe operation in many industrial plants. The scope and types of inspections are well described in the plant inspection plan, where requirements are based on the detection and evaluation of the different types of defects and their size. The detection and measuring of defects are needed due to legal requirements and to ensure the plant safety. These defect measurements are analyzed also to obtain trends and predictions, which are used as guidelines for maintenance plans or even plant modifications.

The inspection of pipelines and tanks is a very relevant use case in oil and gas plants maintenance. Corrosion is the most common failure in pipes and it could occur inside a pipe, caused by the transported medium, or it could be found outside the pipe due to the surrounded environment, or atmospheric conditions, like rain and humidity. Two of the major problems produced by corrosion are the decrease in wall thickness and the cracking of the pipe. These types of defects are being measured nowadays using sensors that require contact. Ultrasonic inspection is a well-established and standardized method for corrosion detection, wall thickness measurement, and sizing of the defect. Eddy current testing is also applied [10]. Ultrasonic testing (UT) requires that the sensor is coupled to the component being inspected through a couplant. Eddy current testing does not require the couplant, but it needs to be attached to the scanned surface in order to omit lift-off effects.

Plant inspection has been traditionally performed by technicians accessing to the specific inspection points. The inspection points are often located at inaccessible heights and the operator needs to use man-lifts, cranes or scaffolds to have access to their locations. The use of rope-access techniques has been generalized in the oil and gas sector [11] after the success of the first projects in offshore platforms in the North Sea in the 1970s. In onshore plants, rope-access allows reducing inspection costs by up to 90% when compared to traditional methods (scaffolding, lifting platforms, etc.). In offshore plants, rope-access is the only technically and economically viable solution. Also, placing the inspection transducer at certain positions and scanning along the pipes omitting flanges, nozzles, holding gaskets is costly and time-consuming. It is estimated that a small-sized refinery with one crude unit requires inspecting by ultrasound technique more than 15,000 points each year and at least 30% of them are in areas of very difficult access. Furthermore, the pipes or surrounding objects could have high-temperature surfaces (up to 400 ${}^{\circ }$C) or contain potentially dangerous materials or fluids, substantially increasing the risk of falls or other incidents.

There is a strong demand in the oil and gas industry to find alternatives to manual inspection in order to reduce risks and costs. One of the first technologies adopted is based on nodes—with sensors, wireless communications, and integrated battery—deployed at selected locations in the plant [1,2]. These sensors can gather measurements very frequently and are suitable for intensive monitoring of critical components. They operate in an unattended way for long periods of time under potentially bad conditions, originating frequent sensor failures. Manual recalibration or substitution of sensors located at inaccessible locations is dangerous and costly. Some methods, such as [12], detect the consistency of the measurements of a sensor comparing its measurements to those from nearby sensors and detect the sensor it is faulty or badly calibrated. Sensor-based technology provides punctual measurements and is suitable for intensive monitoring of few or small critical components but it is not ideal for covering a full oil and gas plant.

Recent solutions using robots with magnetic wheels have been applied [3]. They can solve some inspection problems, but usually, have low accessibility to pipes at height. These robots need heavy magnetic wheels, which are difficult to be detached and are valid only for pipes with ferromagnetic surfaces. Other solutions are robots navigating inside the pipes [13]. They have similar accessibility problems and require having the pipe out of service during the inspection. Moreover, robot malfunctioning may result in pipe blockages. Accessibility is the main problem of robot technologies particularly for inspecting locations at a certain altitude and isolated from nearby structures.

Aerial robot manipulation is growing quickly in the last years towards a stronger position in robotics. Two DoF robotic arms installed onboard aerial vehicles have performed grasping and deployment of objects [4] or turning valves [5]. Multirotor and helicopter aerial robots with onboard six DoFs robotic arms have been developed [14,15]. Aerial manipulators were also been used for assembly of 3D structures [6].

One of the main research challenges in aerial robotics manipulation is to keep stability during physical contact. In [7] it is shown how keeping contact when the robot is below the contact point improves stability. Compliant articulated robotic arms mounted on board multirotors have been analyzed [8]. In [16] Delta manipulators were integrated on a side of the aerial robot and the stability problem was studied, making the interaction with the environment compliant. The aforementioned robots are capable to contact at points located either under or at the front of the robot. In [17] an aerial vehicle that operates both at the bottom and front is presented. In [18] aerial manipulators capable to operate in any direction were presented. Stable contact operations have been achieved by a pushing trirotor in [19] or, a quadrotor in [20]. In [21] a long rigid tool exerted forces against a surface. A compliant robot that preserved stability during collision is presented in [22]. Also, new aerial robots capable of 6D physical interaction [23] and performing collaborative transportation [24] were also presented. The great majority of the above robotic solutions were tested only in the laboratory or under very controlled conditions. Although many advances and RnD activities have been performed in aerial manipulator systems in the last years, in general, these systems do not have the necessary robustness and are still far from their everyday application in industrial conditions.

Some drones are conceived as robust solutions for the industry, such as [25] or [26], but they are not able to perform contact inspections. The first aerial vehicles for contact inspections in industrial plants were announced in 2018. Two good examples are [27,28]. Existing solutions can contact only vertical surfaces—to the best of our knowledge only [27] can contact also inclined surfaces. In existing systems, the pilot guides the UAV to the surface until it contacts the surface. Once the contact has been established, the contact point cannot be moved by the pilot. Hence, if the pilot did not contact exactly at the desired location, he has to repeat the contact operation. Hence, these systems have very low accuracy selecting the inspection points—it depends on the pilot expertise, and cannot perform continuous inspections such as B-scan or C-scan, which are measuring techniques related to the capture of data while moving continuously over the surface [29].

The main advantages of AeroX over existing systems can be summarized as:

- It can perform contact inspection on surfaces at any orientation including vertical, inclined, horizontal-top, and horizontal-bottom.
- During contact it has fully-autonomous GNSS-free navigation. It flies with uninterrupted contact keeping steady its position w.r.t. the surface contact point using only its internal sensors. The inspector can move the wheels of the end-effector with uninterrupted contact enabling accurately selecting the inspection point.
- It can be operated very easily by two persons; the pilot and the inspector, who do not need to be aware of the aerial part of the system while operating the movement and coupling of the contact sensors.
- AeroX has robust and stable flight capabilities. The aerial robot controller is able to efficiently compensate perturbations thanks to its design, which transmits the contacting forces to the robot center of mass and allows small movements of the aerial part of the robot in every DoF to absorb disturbances such as wind. AeroX has an eight-tilted rotor configuration which provides high maneuverability, agility, and robustness to rotor failure.
- Its end-effector can be easily interchanged, giving new inspection capabilities to the robotic system.

## 3. The Aerial Manipulator Solution

The design and development of AeroX were based on requirements driven by the industry inspection end-user needs for giving successful solutions to the industry. The oil and gas industry imposes very tough requirements. The operation of the robotic solution should be robust under nominal working conditions at heights with potential strong wind gusts. Besides, the proposed aerial robotic system should be integrated with the current maintenance operations in many industries and should be easily operated by the personnel that is currently involved in industrial plant inspections, which often have low training in robotics. The main requirements considered in the design of AeroX can be summarized in the following:

- It should keep a sensor in steady physical contact at a point on the surface where a measurement is going to be taken.
- It should mount a variety of physical contact sensors such as Eddy-current sensors or ultrasonic sensors that measure thickness and corrosion, among others.
- The aerial platform should have fast reactivity and controllability in order to fly close to obstacles in constrained environments with wind gusts.
- It should be easy to be operated for the personnel currently involved in industry inspection with low training.
- Its operation should be easily adaptable to the specific inspection procedures currently used in industries.
- The robotic system should be very robust and reliable for everyday operations in industrial settings.

**Figure 2:**
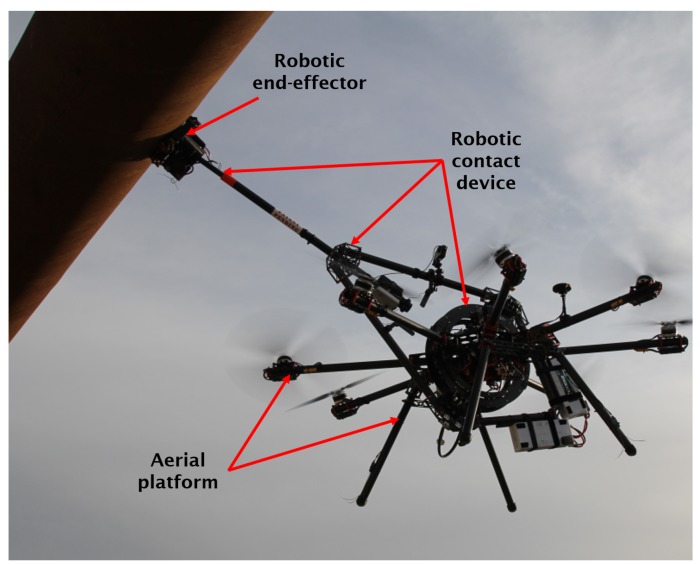
The AeroX robot in physical inspection of a pipe during a validation experiment.

The proposed aerial robotic system—the AeroX robot—has three main components, see Figure 2:

- The aerial platform. Its design enables applying on the surface the contact forces required for physical inspections. The aerial platform is endowed with tilted rotors, which, as will be described later, increases its stability and reactivity to compensate perturbations.
- The robotic contact device. It is responsible for providing the capability of steady contact with surfaces. It is a mechatronic device with six DoF and has the robotic end-effector at its end. Due to its efficient design, surface contact forces are transmitted to the center of mass (CoM) of the aerial robot which enables their efficient and effective compensation.
- The robotic end-effector. It is located at the end of the robotic contact device and is endowed with wheels for moving on the surface under inspection. It integrates the sensors to be used for inspection and also additional sensors to facilitate operation, e.g., a camera for helping the operator identify the defects under inspection. A variety of robotic end-effectors can be mounted on the robotic contact device in order to use different sensors, change the motion on the surface of the robotic end-effector or even change the type of docking to the surface. An end-effector with four wheels, an ultrasound sensor, and a gel injection device is presented in this paper.

The operation of AeroX has two main modes. In the free-flight mode, the pilot guides the aerial robot (in manual or assisted way) to the element to be inspected and moves the robotic contact device to the selected inspection point until the robotic end-effector is in contact with the surface. As soon as the contact has been performed, the contact-flight mode starts, the pilot activates the fully-autonomous stabilization mode, which keeps the aerial vehicle steady w.r.t. the surface contact point with uninterrupted contact using only the measurements from the robot internal sensors, see Section 4.1. The inspector teleoperates the movement of the wheels of the robotic end-effector on the surface. As a result, when the inspector moves the wheels of the end-effector, the surface contact point changes and the aerial vehicle moves to keep its position steady w.r.t. the surface contact point. Hence, the aerial robot follows the end-effector commanded by the inspector. Some videos of the operation of AeroX can be found in Section 6.3.

## 4. Main AeroX Components

This section summarizes the main components of AeroX including the aerial platform, the robotic contact device, and the robotic end-effector.

### 4.1. Robotic Contact Device

**Figure 3:**
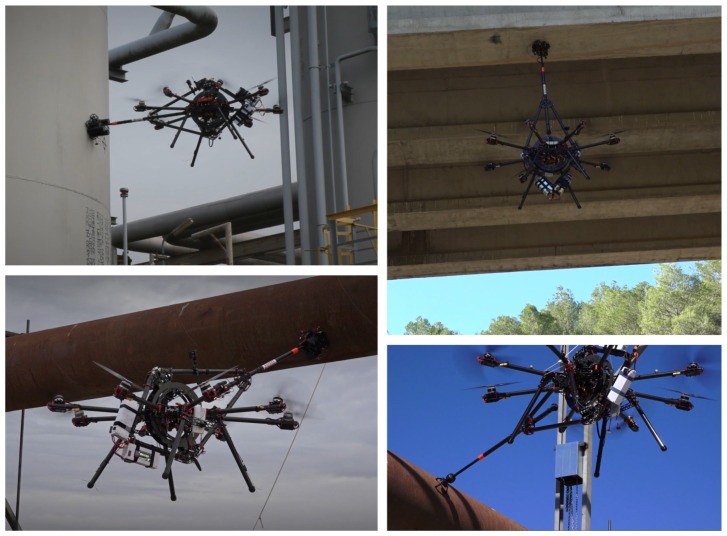
AeroX in contact inspection of surfaces with different positions orientations in different outdoor experiments and with different end-effectors: inspecting pipes (**bottom-left** and **bottom-right**), horizontal ceilings (**top-right**) and vertical surfaces (**top-left**).

One of the main components of the proposed aerial robotic system is its novel robotic contact device, whose patent has been granted at the beginning of 2018 [9]. Due to its mechanical design and integration to the aerial platform, all the surface contact forces are transmitted to the CoM of the aerial robot, which enables simplifying the stabilization and control of the aerial vehicle. With the robotic contact device, the aerial robot is capable of absorbing perturbations, keeping a robust and stable contact perpendicular to the contacted surface. The main characteristics of the robotic contact device are:

- Its mechanical design and integration with the aerial platform enable inspecting surfaces with different positions and orientations such as vertical, horizontal and inclined surfaces, see pictures from outdoor experiments in Figure 3.
- The robotic contact device transmits all forces directly to the CoM of the aerial vehicle, simplifying its stabilization and control.
- All the joints of the robotic contact device are equipped with shock absorbers, which increase perturbation rejection and surface compliance during contact.
- The joints of the robotic contact device are equipped with internal sensors. During contact-flight their measurements are used to estimate and control the relative position of the aerial robot w.r.t. the contact point using only its internal sensors and without any external positioning system or sensor.

**Figure 4:**
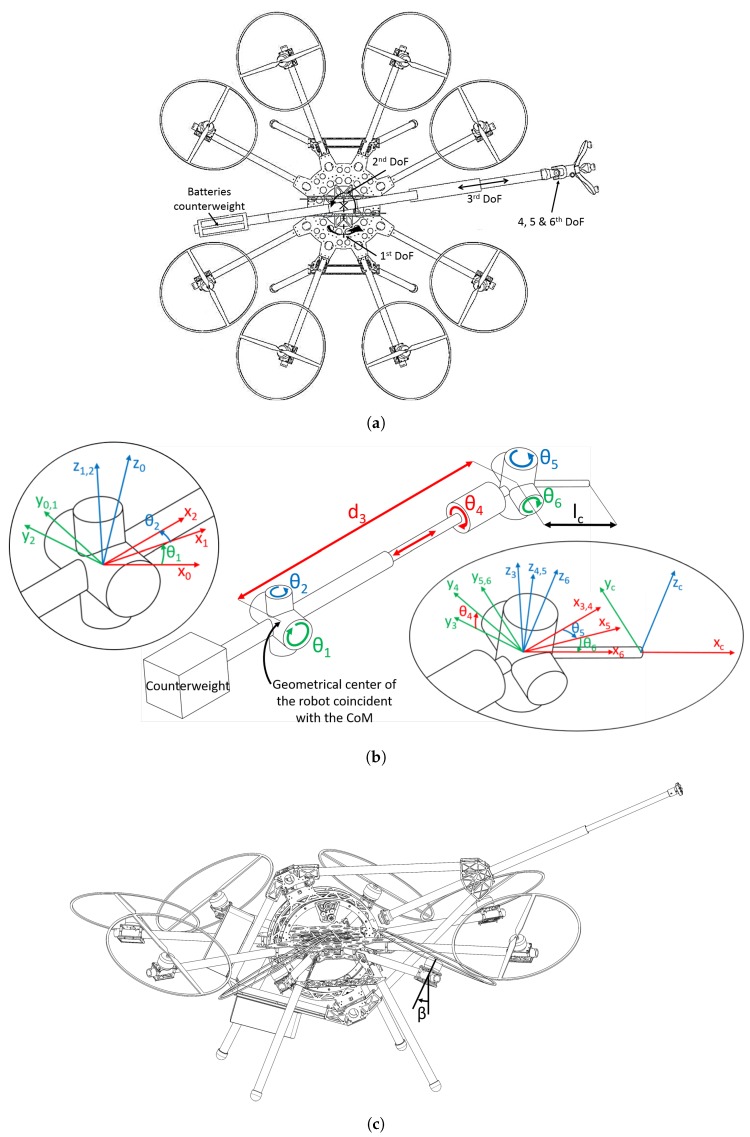
Different schemes of the proposed aerial robot showing its kinematic representation. (**a**) Drawing of AeroX. Its size is 170 × 230 cm. (**b**) Scheme of the robotic contact device showing its six joints and the reference systems for each DoF. (**c**) Drawing of the aerial robot. The tilted-rotor configuration can be noticed. Each rotor is alternatively tilted a fixed angle β=1/6 rad around its motor boom.

Figure 4b shows a scheme of AeroX with the six joints of the robotic contact device, which provides six DoFs. The batteries of the robot are located (as a counterweight) at the other side of the robotic device in order to equilibrate the torque produced by the weight of the end-effector. The first three joints of the robotic contact device ($\theta_{1},\theta_{2}$ and $d_{3}$) follow a polar robot configuration. The first two joints are rotations around the center of the aerial vehicle. Giving positive angles to $\theta_{1}$ would move the end-effector to the top of the body of the aerial vehicle. The second, $\theta_{2}$, is a rotation that would move the end-effector to the left side of the aerial robot body. The third joint produces a linear motion which axis goes through the center of mass (CoM) of the aerial vehicle, which is located exactly at the geometrical center of the aerial robot body, see Figure 4b. The joint variable $d_{3}$ is defined as the distance between the robot CoM and the axis intersection of the last three joints $\theta_{4},\theta_{5}$ and $\theta_{6}$.

The last three joints are rotations located close to the robotic end-effector and enable orientating it perpendicularly to the contact surface. The rotation axes of these last joints coincide at the same point, as shown in Figure 4b. The distance between this point and the surface contact point is denoted as $l_{c}$.

Each of the three first joints of the robotic contact device is equipped with a sensor. During contact, their measurements are used to estimate at a high rate (100 Hz) the position of the aerial robot w.r.t. the surface contact point. This relative position together with the robot orientation measured with the inertial measurement unit (IMU) is used for high-frequency stabilization and hovering, enabling fully-autonomous manipulation operations without relying on any external positioning system.

During contact-flights, all joints of the robotic contact device turn into a passive mode (zero torque mode) enabling the aerial vehicle to move freely without friction coming from the joints. Hence, the CoM of the robot receives only a linear force from the surface contact and does not receive torques that could destabilize the aerial robot. The integration of the robotic contact device with the aerial platform is such that during contact-flights the robot can change its pose w.r.t. the robotic end effector a few centimeters in any direction and a few degrees in any orientation, 20 cm and 10 degrees in the prototype used in the experiments. This provides the aerial vehicle capability to reject flight perturbations (e.g., due to wind gusts) during the contact-flight without changing the contact point.

Using the joint sensors to estimate the aerial robot pose has clear practical advantages for industrial inspections. Firstly, it enables very high-rate and accurate pose estimations using robust and simple mechanical joints and onboard sensors. The accuracy of the resulting position is similar to that from common high-precision commercial off-the-shelf (COTS) real-time kinematic (RTK)-GPS but with significantly higher rates that enable real-time control and also the advantage that it is valid in environments with poor or non-existent GNSS coverage.

In the following, we obtain the expressions used to compute the position of the aerial robot w.r.t. the contact point using only its internal sensors. The CoM of the aerial vehicle is located at the geometrical center of the aerial robot body, where the axes $\theta_{1}$ and $\theta_{2}$ coincide, see Figure 4b. A frame is defined for each joint of the robotic contact device, see Figure 4b. The aerial robot coordinate frame, whose origin is at the robot CoM, is denoted as frame 0. The relative pose of the surface contact point w.r.t. frame 0 is expressed as:

(1) $${}_{6}^{0}T={\;}_{1}^{0}T{\;}_{2}^{1}T\;\ldots {\;}_{6}^{5}T,$$

where ${}_{Y}^{X}T$ is the transformation matrix from frame Y to frame X.

In many cases $l_{c}$ can be considered negligible due to the low size of the end-effector, so in this case the origin of the frame of joint 3, see Figure 4b, can be taken as the surface contact point. The transformation between the origin of the frame 3 and the aerial robot frame can be expressed as:

(2) $${}_{3}^{0}T=\left[\begin{array}{cccc} c\theta_{1}c\theta_{2} & -c\theta_{1}s\theta_{2} & -s\theta_{1} & c\theta_{1}c\theta_{2}\;d_{3}\\ s\theta_{2} & c\theta_{2} & 0 & s\theta_{2}\;d_{3}\\ s\theta_{1}c\theta_{2} & -s\theta_{1}s\theta_{2} & c\theta_{1} & s\theta_{1}c\theta_{2}\;d_{3}\\ 0 & 0 & 0 & 1 \end{array}\right],$$

where sθ stands for sin(θ) and cθ stands for cos(θ). All operations in each component of ${}_{3}^{0}T$ are trigonometric, hence all involved forces and torques are transmitted directly to the CoM.

The first three elements in the last column of ${}_{3}^{0}T$ represent the relative position of the surface contact point w.r.t. the aerial robot frame expressed in the robot frame. It will be called $SCP_{local}$. During the flight, the orientation of the aerial robot (and of its reference frame) might be different from the Earth reference frame. Hence, we need to correct $SCP_{local}$ in order to express this translation in Earth axes. The orientation of the aerial robot reference frame w.r.t. the Earth axes can be measured accurately and with a high rate (200 Hz) with the robot IMU and magnetometer. With their measurements, we compute the direct cosine matrix (DCM), a rotation matrix that transforms from the aerial robot frame to a frame whose origin is the robot CoM but has Earth axes. Hence, it is easy to note that the relative position of the robot CoM in Earth axes w.r.t. the surface contact point can be computed very efficiently as follows:

(3) $$pos_{Robot}=-DCM\;\;\;SCP_{local}.$$

Notice that the pose of the aerial robot w.r.t. the surface contact point is fully defined by $pos_{Robot}$ and DCM. Hence, the adopted method computes very efficiently and at a rate of 100 Hz the robot pose w.r.t. the surface contact point using only internal sensors. This provides high autonomy, which is of interest for operation in unstructured scenarios such as factories. This robot pose estimation is used for fully-autonomous robot stabilization and control during contact-flights, as described in Section 5.1.

### 4.2. The Aerial Platform

The controllability and reactivity to fly close to obstacles and withstand wind gusts, maneuverability and agility to fly in constrained industrial environments and the robustness for everyday operation have been the main characteristics in the design of the aerial platform.

The proposed aerial vehicle has eight 2 kW motors (KDE5215XF-220), providing a maximum take-off weight of 25 kg. The motors are set such that the robotic contact device can rotate around the vehicle CoM without colliding with the propellers. Each set of motor-propellers is alternatively tilted 30 degrees around the motor boom, see Figure 4c. The tilting of the motors are fixed and cannot be controlled. The actuation variables that can be controlled are each of the eight motor desired forces. Tilted rotors enable independent control of the six DoF of the vehicle adding the capability to control the linear lateral forces, providing full control of the aerial platform during hovering, highly improving the aircraft controllability [23]. Furthermore, as demonstrated in [30], six tilted propellers are sufficient to be able to control the six DoF of the vehicle and ensure control of four DoF in case one motor fails. The adopted configuration with eight motors was selected to overcome the failure of one motor keeping the full robot six DoF controllability. This makes this aerial vehicle the first one of its class, this feature being an important step to its future industrialization.

The aerial platform design and its hardware elements integration have been carefully implemented for distributing the weight in order to keep the CoM of the robot at the geometric center of the aircraft. Besides, as described in Section 4.1, the integration of the robotic contact device is such that the surface contact forces are transmitted to the CoM of the aerial vehicle, which improves stability and controllability during contact-flight mode.

#### 4.2.1. Control Allocation

AeroX control architecture is based on a typical multirotor control architecture but adapted to exploit the maneuverability advantages of its six DoF tilted-rotor configuration. Multirotor control architectures compute *W*, which in standard multirotor configurations, include only the vehicle torques and throttle force ($F_{z}$, the linear force in the Z robot body axis). Typical control architectures also include a module called the mixer which transforms *W* to $f_{m}$, a vector that contains the forces to be exerted by each individual rotor. The relation between $f_{m}$ and *W* is given by *A*:

(4) $$W=A\;f_{m}.$$

The six DoF tilted-rotor configuration of AeroX enables controlling the three vehicle torques and throttle force, and also controlling lateral forces (linear forces $F_{x}$ and $F_{y}$ in the X and Y robot body axes), which enables lateral motion control without changing the robot attitude involving significant maneuverability advantages critical for navigating in constrained scenarios or rejecting flight disturbances, e.g., due to wind gusts or surface contact forces. In our system, the *W* computed by the AeroX control system includes the three vehicle torques and also the three vehicle forces ($F_{z}$, $F_{x}$ and $F_{y}$). The approach adopted was to modify the mixer in order to add control of $F_{x}$ and $F_{y}$. We modeled the matrix $A\in R^{6x8}$ using the geometry of AeroX. The *A* matrix obtained for AeroX was:

(5) $$A=\left[\begin{array}{ccc} \vartheta_{1} & \ldots & \vartheta_{8} \end{array}\right]$$

(6) $$\vartheta_{1,3,5,7}=\left[\begin{array}{c} -U_{n}\\ -V_{n}\\ c\beta \\ V_{n}\;\;l_{z}+U_{n}\;\;d_{m}-s\gamma_{n}\;\;l\;\;\beta \\ -U_{n}\;\;l_{z}+V_{n}\;\;d_{m}-c\gamma_{n}\;\;l\;\;c\beta \\ -s\beta \;\;l-c\beta \;\;d_{m} \end{array}\right];\;\;\;\;\;\vartheta_{2,4,6,8}=\left[\begin{array}{c} U_{n}\\ V_{n}\\ c\beta \\ -V_{n}\;\;l_{z}+U_{n}\;\;d_{m}-s\gamma_{n}\;\;l\;\;c\beta \\ U_{n}\;\;l_{z}+V_{n}\;\;d_{m}-c\gamma_{n}\;\;l\;\;c\beta \\ s\beta \;\;l+c\beta \;\;d_{m} \end{array}\right],$$

where *n* is an index that refers to each of the eight motors of AeroX, β the rotation angle of each motor around its boom, and γ is the angle of each motor arm w.r.t. the *X* axis in the aerial robot frame 0 measured on the *XY* plane. For brevity, we used $U_{n}=s\beta \;\;s\gamma_{n}$ and $V_{n}=s\beta \;\;c\gamma_{n}$. $l_{z}$ is the height of the center of the propeller with respect to the CoM of the aircraft, $d_{m}$ the drag force constant of the motor + propeller system and *l* is the shortest distance in the robot body *XY* plane from the CoM to the center of the propeller.

Since *A* is not square, using its pseudo-inverse ($H=A^{T}/\left(AA^{T}\right)$) it is simple to compute the forces $f_{m}$ that must be exerted by each rotor of AeroX in order to provide the forces and torques *W* commanded by the controller:

(7) $$f_{m}=H\;W.$$

However, the pseudo-inverse could lead to problems if, after applying (7), any of the components of $f_{m}$ turn to be over or under the admissible forces the motors are able to generate. This problem is avoided by, firstly, saturating each input and output of the controllers (presented in Section 5.1) which results in admissible forces and torques (*W*) that could be safely transformed to $f_{m}$, and secondly, with a saturation module executed after all the controllers which avoids the non-possible motors forces. Meaning that if the first measure fails, the emergency saturation will act, avoiding unstable behaviours of the aerial platform during the time it is active.

The emergency saturation is performed using a hierarchical method, which performs the saturations step by step according to their importance. In this way, $F_{z}$ is considered the most important DoF of the aerial platform, followed by the roll and pitch stabilization torques $T_{x}$ and $T_{y}$, then $T_{z}$, needed for stabilizing the yaw angle, and finally, the horizontal forces $F_{x}$ and $F_{y}$, which are considered to be the less important stabilization forces for safely controlling the flight of the robot. For each step, the module uses their corresponding columns of the matrix *H* to calculate which is the maximum admissible force or torque that could be applied. For each step calculation, the previous hierarchical calculations of each motor force are taken into account.

Furthermore, incremental maximum motor forces are considered for each step. In our specific configuration the first step ($F_{z}$) saturates at the 80% of the maximum force of each motor ($f_{m}$), the second step ($T_{x}$ and $T_{y}$) takes into account a maximum force of a 90%, 95% for $T_{z}$, and 100% for $F_{x}$ and $F_{y}$. This method allows us to keep some margin for the subsequent saturation steps in the hierarchy, leaving a few control authority to all the steps in case some of them saturate.

This approach enables easily adapting widely-tested standard multi-rotor control structures to aerial platforms with specific configurations. The transformation from *W* to $f_{m}$ and the subsequent saturations were implemented in the mixer and saturations module that is described in Section 5.1.

### 4.3. The Robotic End-Effector

The robotic end-effector is located at the end of the robotic contact device. Its hardware components were carefully selected taking into account their weight in order to reduce the unbalance, which produces a torque over the center of mass. As described in Section 4.1 unbalance is partially solved by placing the robot batteries as counterweights. During contact flights, the robot controller hovers, keeping its pose steady w.r.t. the surface contact point keeping uninterrupted contact. This enables taking surface measurements that require keeping the contact steady for several seconds, e.g., ultrasonic measurements. The robotic end-effector is equipped with wheels, which motion can be teleoperated. When the wheels move, the surface contact point moves but the relative pose of the robot w.r.t. the contact point is kept steady: AeroX moves following the motion of the end-effector. Hence, the inspector can easily change the contact point completing the inspection mission on that surface.

**Figure 5:**
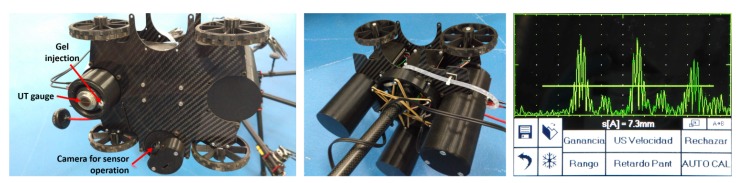
(**left**) and (**center**) Developed robotic end-effector validated in the field experiments equipped with four independently controlled wheels, a UT gauge, a gel injection device and a small camera for sensor-surface contact monitoring. (**right**) Screenshot of the UT sensor interface showing measurements taken during contact with a surface of 7.3 mm of thickness.

In our system, the aerial vehicle creates a force against the surface in order to keep contact. The end-effector uses standard lightweight 3D printed rubber wheels that avoid sliding (Figure 5). The wheels have mechanisms that provide some flexibility so that the end-effector can accommodate better rotation errors and surface inconsistencies. A variety of robotic end-effectors can be mounted on the robotic contact device in order to use different sensors, change the motion of the robotic end-effector on the surface or even change the type of docking to the surface. The main components of the end-effector used in the validation experiments (see Figure 5) were:

- The ultrasonic sensor (UT gauge). It is used for measuring the thickness of metal surfaces. The sensor model selected is a dual element UT gauge for thickness from 3 to 60 mm and it can be controlled remotely using its ISONIC utPod electronics (see Figure 5), which is connected to a computer running their proprietary software. The application is accessible from the ground using the common wireless access point.
- The gel injection device. The selected sensor needs couplant gel to work correctly. An injection gel system with a small gel container and a motor for gel application was installed.
- A system for sensor placing and retraction. In order to avoid strong hits between the sensor and the surface, this mechanism is used to retract and deploy the sensor. The sensor is kept retracted (protected) and it is only deployed while the end-effector has good contact with the surface.
- A small camera. It was located very close to the ultrasonic sensor to visually check the contact between the sensor and the surface. In addition, another camera was installed on the robotic contact device to give the inspector a wider view of the operation.
- Micro-motors: Each end-effector wheel has a micro-motor with a quadrature encoder that can be teleoperated and controlled in velocity. Each motor can be controlled independently enabling moving and rotating in any direction. It has been validated experimentally that the end-effector can operate as expected even with only two wheels in contact with the surface.

## 5. Control and Operation of the Inspection System

### 5.1. Control Architecture

**Figure 6:**
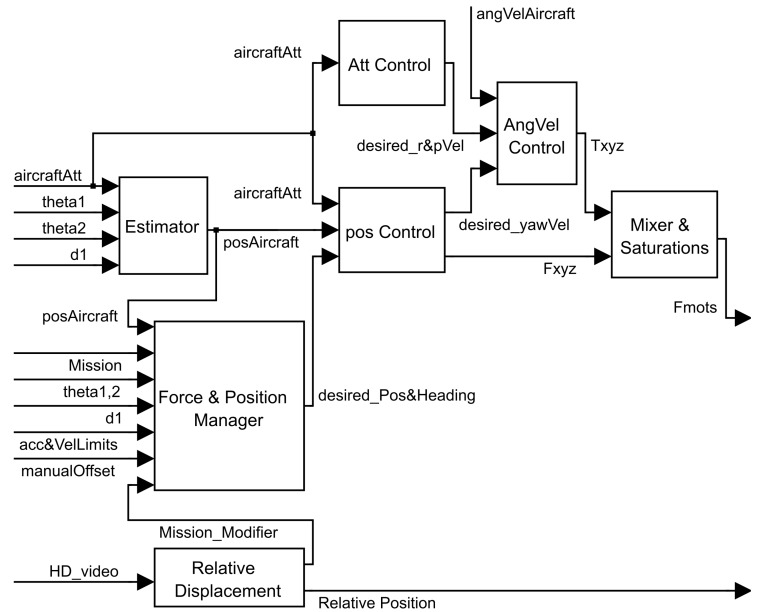
Control architecture of AeroX.

The control architecture of AeroX is used to keep steady the robot position w.r.t. the surface contact point during contact flights. It is shown in Figure 6. The control architecture is based on that of a standard multirotor but modified to exploit the advantages of its six DoF tilted-rotor configuration. In standard multirotor control architectures, the three main controllers (position, attitude and angular velocity) are connected in cascade. A module called mixer transforms the output of the cascade—the three torques and the throttle $F_{z}$ in the robot body axis *Z*—to the forces to be exerted by each rotor. The mixer in the AeroX control architecture enables controlling the three torques, the throttle $F_{z}$ and also linear lateral forces $F_{x}$ and $F_{y}$ in the robot body axes *X* and *Y*. This enables lateral motion control without changing the robot attitude, which is the main advantage of tilted-rotor multirotor over standard multirotor configurations. Furthermore, the control architecture has been reorganized and simplified removing the position control from the cascade and executing it in parallel with the attitude and angular velocity controllers, see Figure 6. The AeroX control architecture also includes extra modules to compute the relative pose of the aerial robot w.r.t. the surface contact point.

Below the main modules in the AeroX control architecture are presented:

- Estimator. It computes the estimate of the relative position the aerial vehicle w.r.t. the surface contact point as detailed in (3).
- Force and position manager. This module computes the desired heading and relative position of the aerial vehicle w.r.t. the surface contact point. Its output is sent to the position controller. The desired position and heading depends mainly on: (1) the desired force to be applied against the surface; (2) the robot relative position w.r.t. the surface contact point and; (3) the desired mission configuration (e.g., desired force against the surface).
- Position controller. This module computes the linear forces ($F_{x}$, $F_{y}$ and $F_{z}$ in robot body axes) of the aerial robot using the position error of the robot. In contact flight, the objective is to keep steady the relative position of the robot frame w.r.t. the surface contact point. The reference is the desired relative position computed by the force and position manager and the current robot relative position is computed by the estimator. This module also computes the yaw velocity using the heading error (the offset with respect to the Earth’s north).
- Attitude controller. The roll and pitch velocities are computed using the errors of the roll and pitch angles. As AeroX can perform lateral movements without changing attitude, the roll and pitch input references are always set to 0 in order to improve the robot stability and simplify the control.
- Angular velocity controller. This module computes the desired torques of the aerial vehicle using the error between the angular velocities commanded from the attitude controller and the velocity measurements estimated by the onboard sensors.
- Mixer and saturations. This module implements the control allocation detailed in Section 4.2.1. It transforms *W*, the output of the whole controller (the three torques, the lateral forces $F_{x}$ and $F_{y}$, and $F_{z}$), to $f_{m}$, the forces to be exerted by each rotor. For doing so, it takes into account the achievable forces by each of the eight motors, saturating the desired *W* in a controlled hierarchical way (see Section 4.2.1).
- Relative displacement. This module implements the method described in Section 5.3 and computes the relative displacement of the end-effector on the surface under inspection. The output of this module can be used as input of the force and position manager, which uses it to compute the desired heading of the aerial vehicle.

### 5.2. Inspection Procedure

The operation of AeroX in the inspection process involves two human actors: the pilot of the aerial robot and the remote inspection operator. The operation of each actor is independent of one another. This approach benefits from their complementary expertise preventing that they disturb one another with the details of their tasks. It should be noted that involving two human actors is the most suitable solution to operate AeroX: the pilot focuses its attention only on the flight safety and the inspector is fully focused on the operation of the end-effector and the fulfillment of the inspection plan. Furthermore, most of the countries’ regulations require a certified pilot for a UAV and a certified inspector for the inspection.

The inspection procedure starts with an inspection plan generated by the responsible of the industrial plant. Following that guideline, the field inspector decides where and how to perform the inspection. The operation of the system is as follows. The pilot guides the aerial robot in free-flight mode to the element to be inspected and moves the robotic contact device to the selected surface. The inspector has no intervention while in free-flight mode. As soon as contact is performed, the pilot switches to contact-flight mode, which keeps steady the pose of the robot w.r.t. the surface contact point in a fully-autonomous way. During contact flights there is no intervention of the pilot: he only supervises the status and correct performance of AeroX or modifies flight parameters if required.

During contact-flight mode, the end-effector exerts a force on the surface enabling uninterrupted contact. When the inspector actuates on the end-effector wheels, the surface contact point changes while the robot pose w.r.t. the contact point is kept steady: AeroX follows the motion of the end-effector. The inspector can teleoperate the motion of the end-effector on the surface, can change the inspection sensor parameters, and can visualize the operation using the onboard cameras. The inspector can perform the inspection on the desired points according to the plan. When all inspection points have been analyzed, or when another surface must be inspected, the pilot deactivates the contact-flight mode, takes control of AeroX and guides it to the following surface to be inspected.

**Figure 7:**
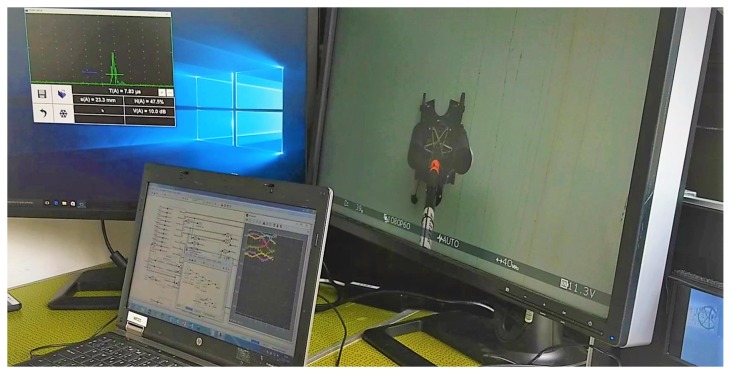
Main three screens visualized by the inspector during operation. On the right screen an onboard camera shows the end-effect and the surface under inspection. The screen on the left is used to manage and visualize the inspection sensors: an ultrasound measurement during this flight experiment is shown. The laptop shows the telemetry and status of AeroX.

AeroX is suitable to perform: (1) A-scan punctual inspections, e.g., specific points at inspector selected areas; (2) B-scan inspections, the points measurements are continuously taken along a surface with only linear positioning information and; (3) C-scan inspections, a continuous scan on the two dimensions of a surface. The validation experiments shown in Section 6 are A-scans. Figure 7 shows the main screens visualized by the inspector during the operation. The onboard camera (at the right in Figure 7) was useful to identify the defects to be inspected. However, the inspector was rather inaccurate estimating distances between inspection points when visualizing only onboard cameras. To cope with this, AeroX includes a vision-based relative localization system that provides the inspector with estimates of the relative displacements of the end-effector on the surface under inspection.

### 5.3. Vision-Based Relative Localization Assistant

Its objective is to provide the inspector with estimates of the motion of the end-effector on the surface to assist the inspection operation. Odometry techniques that estimate motion using the encoders of the end-effector wheels are too inaccurate in our problem due to the significant wheel slippage on many surfaces that can be found in industrial settings. A more accurate visual-based motion estimation method was developed. The developed technique receives as input the images captured by the camera attached to the robotic contact device. As the end-effector moves, it always appears at the same position in the images whereas the surface that is seen in the images moves. The developed algorithm detects visual features (small stains, marks, and irregularities) on the surface and computes their relative displacement from one image to another, which are used to estimate the relative motion of the end-effector on the surface.

**Figure 8:**
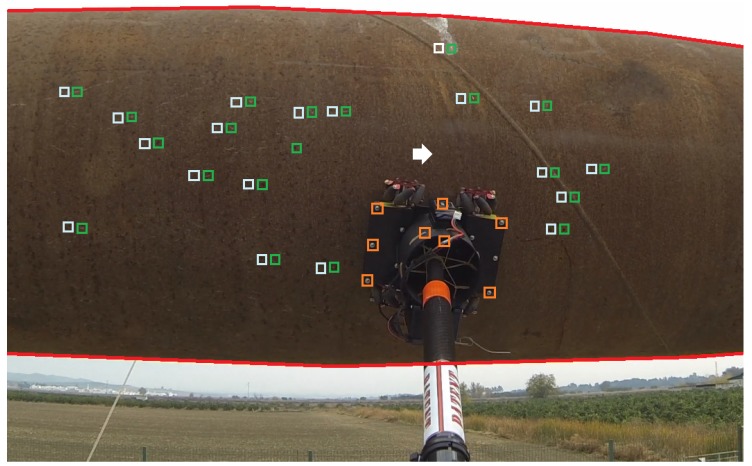
Picture of the on-board camera used for the visual-based relative localization system. The green rectangles are used to mark the SURF features in $FS_{t}$ that were correctly matched—after executing RANSAC—with features in $FS_{t-1}$,—marked with white rectangles. The matchings (of the end-effector) filtered out by RANSAC are marked with red rectangles. A small white arrow illustrates the relative displacement between the end-effector and the pipe between t−1 and *t*.

The developed algorithm executes the following image processing steps. First, the internal camera calibration and lens distortion parameters are used to correct the image distortions. Next, the region of interest (ROI) where the surface is seen in the image is detected and segmented, avoiding the image background, e.g., the sky as in Figure 8. For this, the edges of the surface are detected. A Sobel edge detection operator is applied to enhance the edges in the image. A Hough transform is then used to detect the main horizontal and vertical lines in the image. These lines represent the edges of the surface under inspection. They are used to identify the ROI where the surface under inspection is seen in the image. If the image has no background and only the surface is seen, no edges will be detected: the ROI is the full image.

The next step is the extraction of visual features in the ROI at each image. We used SURF features due to their high sensitivity [31]. The set of features detected at time *t* is denoted as $FS_{t}$. The ROI contains the surface and also the end-effector, see Figure 8. Hence, some features in $FS_{t}$ are features of the surface and, some are end-effector features. The next step is the computation of the matching between features $FS_{t}$ and $FS_{t-1}$ using their SURF descriptor as e.g., in [31]. When the end-effector wheels are activated the surface features will move whereas the end-effector features will be steady, see Figure 8. Matchings between end-effector features are treated as spurious and are filtered out by the RANSAC algorithm [32]. Finally, the filtered feature matchings are used for fitting a transformation matrix model that characterizes the relative translation from t−1 to *t*. The motion in a sequence of images is estimated accumulating the translations between the consecutive images of the sequence. The motion in pixels is finally transformed to meters using the distance to the surface, which is measured by the internal sensors of the robotic contact device.

An image from the onboard camera with some results of the visual-based relative localization technique is shown in Figure 8. The accuracy of the developed vision-based technique has been evaluated in the CATEC indoor testbed using the VICON system as ground truth. The drift measured was lower than 1.8 cm every meter of the end-effector motion, which was considered sufficiently accurate by the end users.

The operation is as follows. When the operator activates the motion of the wheels, the computation of the visual odometry is started. The cumulative motion is shown to the operator, who selects the next inspection point. When the end-effector reaches the next inspection point, the operator deactivates the wheel motion: the computation the visual odometry is stopped.

## 6. Experimental Validation

Different sets of experiments were performed to validate the performance of AeroX. Two types of experiments are presented. First, some experiments in outdoor realistic scenarios—including a refinery in Germany—are described to illustrate the operation and performance of the system. Additionally, its robustness against external perturbations was analyzed in laboratory experiments. Finally, Section 6.3 provides links to different videos showing AeroX validation and robustness analysis experiments.

### 6.1. Validation in Realistic Environments

**Figure 9:**
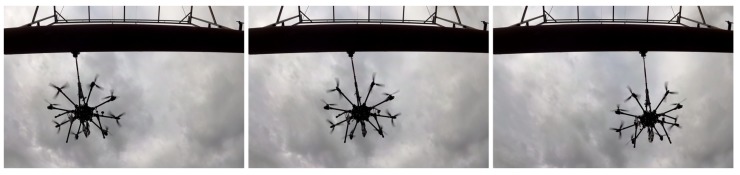
AeroX during contact flight in an experiment performed in Seville in October 2018. AeroX was flying in autonomous mode and the end-effector was teleoperated by the inspector. Images at different times show the motion of the end-effector along the pipe while keeping the relative position of the aerial vehicle.

A total of more than 200 different experiments have been performed in outdoor realistic scenarios. Some experiments were performed in a real oil and gas facility in Germany, see Figure 1 and Section 6.3. Others, were carried out at a testing scenario near Seville with a real pipe of 6 m of longitude and 0.6 m of diameter that was placed 4 m above the ground level, see Figure 2 and Figure 9. In all these experiments AeroX was operated only with a pilot and an inspector non-qualified in robotics. The objective of these experiments was to: (1) validate AeroX as a suitable inspection tool for industry and; (2) validate the AeroX inspection procedure presented in Section 5.2.

Below an experiment performed at the testing scenario near Seville in October 2018 is described as an example of all the different experiments that have been performed in outdoor realistic scenarios. A summary video of that experiment is available at https://youtu.be/nvw0I5ux19U. The inspection procedure described in Section 5.2 is followed. The pilot manually performed the take-off of the aircraft and guided it to the pipe. The contact was performed at time 0:28 s of the video. At that time the pilot switched to contact-flight mode and AeroX kept steady its position w.r.t. the contact point using its internal sensors and not the GNSS measurements. At time 0:35 the inspector moved the end-effector to the right. The images captured by the onboard camera can be seen at time 0:37. At time 0:48 the inspector moved the end-effector to the left. The images taken by the onboard camera can be seen at time 0:59. At 1:32 the pilot deactivated the contact-flight mode, took control of AeroX and landed at 1:43 s.

**Figure 10:**
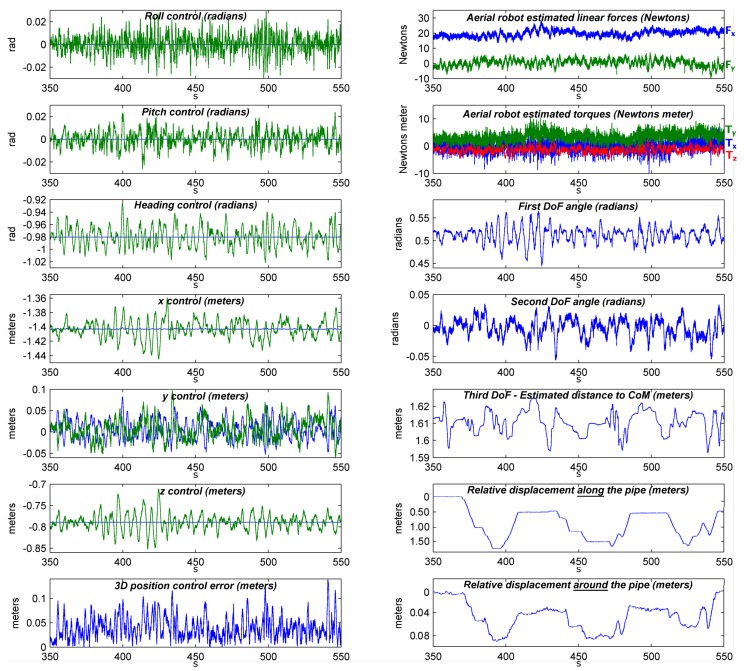
AeroX telemetry during contact-flight in one outdoor experiment performed in the outdoor testing setting near Seville in October 2018. The control responses for attitude and position (blue = reference, green = measured data) as well as the 3D estimated positioning error are shown at the left. The computed linear forces, torques, values of the first joint variables of the robotic contact device are plotted at the right. Linear force $F_{x}$ is in blue while $F_{y}$ is in green. In the torque graph, the torques around axes *X*, *Y* and *Z* are respectively in blue, green and red color. The relative displacement of the robotic end-effector along and around the pipe is presented at the left-bottom.

The resulting telemetry in the above experiment—which took 200 s—is shown in Figure 10. The video only shows a summary of the experiment. Figure 10-left shows the control performance of the aerial robot in terms of position and attitude. Figure 10-left-bottom shows the AeroX positioning error using the proposed controller and the robot internal sensor-based position estimation method developed. The robot RMS positioning control error was of 4.6 cm along the experiment. The maximum instantaneous positioning error measured was 13.8 cm. These results validate the control architecture of AeroX. Figure 10-right-top shows the computed lateral forces ($F_{x}$, $F_{y}$ and $F_{z}$). It can be noticed that the robot is exerting a lateral force ($F_{x}$) of about 20 N against the surface under inspection. Figure 10-right-center shows the readings of the sensors in the three first joints of the robotic contact device. Figure 10-right-bottom shows the displacements of the end-effector, first along and then around the pipe, estimated by the method described in Section 5.3.

The wind measured during this experiment changed between 0 and 3 m per second. The flight of AeroX was unaffected by these wind speeds. Section 6.2 shows that the operation of AeroX is rather robust against significant external perturbations. Also, preliminary outdoor experiments were performed at the outdoor testing scenario to validate the capability of AeroX to make contact—and inspect—vertical and horizontal surfaces.

AeroX was also validated in a real oil and gas plant in Germany in July 2018, whose location is left undisclosed due to confidentiality issues. The main objective was to validate its operation from the inspector and end-user perspectives. The operation of the end-effector was extensively tested on several surfaces with different conditions of dirt, curvature, heights, and accessibility. For all of them, the end-effector was correctly guided by the inspector and UT measurements were taken (see Figure 5 and Figure 7) at all the positions selected by the inspector. The performance of the gel dispenser system of the UT probe was validated. The status of the coupling between the UT sensor and the surface under inspection was also checked using both, the small cameras onboard the end-effector and the real-time A-scan measurements that were visualized in the operator screens.

These aforementioned experiments validated the operation of AeroX and its inspection procedure in industrial scenarios. AeroX can easily fly in constrained industrial scenarios and the inspector can easily select the contact points to be analyzed. AeroX can fly in a fully-autonomous way during contact without any GNSS system or external sensor. The inspection procedure benefits from the expertise of the pilot and of the inspector and clearly separates their roles and operation preventing that they disturb one another.

### 6.2. Robustness Analysis

The operation of AeroX should be robust under nominal working conditions at heights with potential strong wind gusts. A series of experiments have been performed to assess the stability and robustness of Aerox to strong flight perturbations. The experiments were performed in the CATEC indoor testbed, a 15 × 15 × 5 m room equipped with a 20-camera VICON motion capture system [33] capable of providing <1 mm localization accuracy. The VICON system was not used at all in any AeroX component or module, it was used only as ground truth to evaluate its performance.

In these experiments, external perturbations were injected in AeroX during contact in fully autonomous flights. The perturbations were rather high and the experiments were very risky. For safety reasons, a safety rope with nearly zero tension (like a safety belt) was attached on the top of AeroX. This safety rope was not involved at all in AeroX flight: the operation of AeroX and its control system was exactly as described above. In these experiments, AeroX performed exactly the aforementioned inspection procedure. The pilot manually guided AeroX to the surface—vertical in these experiments—in free-flight mode. When the contact was performed, he switched to contact-flight mode and AeroX kept steady its pose w.r.t. the contact point using its internal sensors and not the GPS or the VICON measurements.

**Figure 11:**
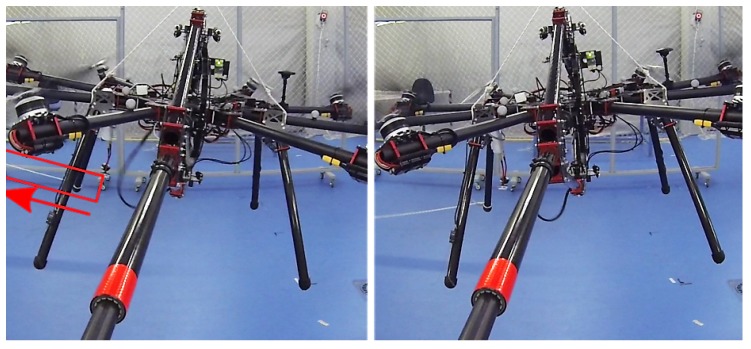
Two images from the video at https://youtu.be/mp4UAuhNHWc taken during the robustness analysis experiments. The safety rope with negligible tension is shown at the top.

The perturbations were injected by pulling a rope attached to the robot landing gear with an approximate force of 100 N, see video at https://youtu.be/mp4UAuhNHWc. Notice that these forces are significantly higher than the perturbations usuallly originated by wind gusts or by the irregularities in the surface contact. Two pictures in this experiment taken from the video are shown in Figure 11. AeroX had clearly different attitudes in both pictures.

**Figure 12:**
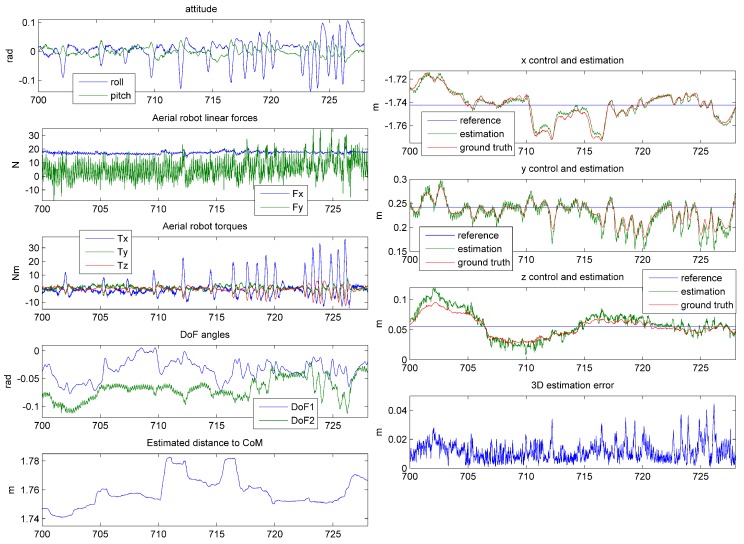
Telemetry of AeroX in fully-autonomous flight during contact in a robustness analysis experiments. The 100 N perturbations injected generated the response of the developed controller, which can be noticed in the robot attitude and torque graphs. On the right, the robot relative position w.r.t. the surface contact point is shown together with the ground truth and the reference values for each axis. The robot position error w.r.t. the reference value in each axis was lower than 6 cm.

The telemetry of AeroX during this experiment is shown in Figure 12. The perturbations induced sudden changes in the attitude of AeroX in axis *X*, which can be clearly noticed in Figure 12-top-left. Figure 12-left shows the torques and linear forces computed by the AeroX controller. To compensate the perturbations the controller actuated with high torques in *X* and lateral forces in *Y*. Figure 12-right shows the relative position *XYZ* of the robot w.r.t. the surface contact point computed by the developed method and also with the ground truth distance measured with VICON. Two conclusions can be reached. First, the robot position estimation error in all axes was lower than 1 cm. The 3D position estimation errors were lower than 4 cm, see Figure 12-right-bottom. The RMS position estimation error in all the robustness analysis experiments was 0.92 cm, which validated the robot relative position estimation method developed. The second conclusion is that the developed controller stabilizes Aerox with a very low error even with high external perturbations. In this experiment the robot positioning error w.r.t. the reference value was lower than 6 cm in each axis.

### 6.3. Video Attachments

Three videos were produced to summarize the experiments performed with the AeroX robot:

- Validation in an oil and gas refinery (https://youtu.be/Hy45WQ3GLcI). This video shows a brief summary of the many hours of operation of AeroX during its validation in an oil and gas refinery in Germany. The inspector guided the end-effector along many different surfaces and took hundreds of ultrasonic thickness measurements at inaccessible elements of the plant.
- Outdoor testing experiments (https://youtu.be/nvw0I5ux19U). This video shows the complete operation of AeroX—from take-off to landing—in our outdoor testing setting in Seville. AeroX was manually controlled in assisted mode during free-flight mode. During contact-flight mode, AeroX flew in an autonomous way without any pilot intervention and the inspector teleoperated the end-effector.
- Robustness analysis experiments (https://youtu.be/mp4UAuhNHWc). This video shows an experiment in the CATEC Aerial Robot testbed. The first part of the video shows how AeroX performs the contact operation on a static surface. During the contact-flight mode, Aerox was in fully autonomous mode. Flight perturbations were injected with a rope attached to the robot landing gear. The second part of the video shows preliminary outdoor tests in which AeroX contacts a pipe in different orientations. Although in these preliminary tests AeroX flew in a fully autonomous way, it was attached to a rope for safety reasons.

## 7. Conclusions

The procedures of the oil and gas industry involve extensive inspections, many of them should be performed at height and using sensors that require to be in contact with the elements to be inspected. The high costs and risks of traditional manual contact inspection using man-lifts, cranes, scaffolds or rope-access have generated in the oil and gas sector a strong need for alternative solutions.

This paper presents AeroX, a novel aerial robotic manipulator that provides physical contact inspection with unprecedented capabilities. AeroX has a semi-autonomous operation. The pilot first guides the robot until performing contact. After contact, the pilot switches to autonomous GNSS-free contact flight and AeroX flies in fully autonomous mode with uninterrupted contact keeping steady w.r.t. the surface contact point. During the contact flight, the inspection operator can move the end-effector wheels on the surface and the aerial robot will follow the motion keeping uninterrupted contact. This can be used for precisely selecting the points to perform A-scan, B-scan, or C-scan inspections. Its operation can be easily integrated into current maintenance programs in many industries. AeroX is operated only by the pilot and by the inspector, each with clear and different roles. The inspector is focused only on the inspection process and does not require any training in robotics. Besides, AeroX has a simple, efficient and robust design that enables its everyday use in fully working conditions. Its tilted-rotor configuration provides high maneuverability, agility to reject flight perturbations and robustness. Its design enables performing contact inspection on surfaces with any orientation, including vertical, inclined, horizontal-top or horizontal-bottom.

AeroX has been extensively tested and validated. Performance analysis experiments carried out in the CATEC indoor testbed shown that AeroX—both in fully autonomous flight as well as flight guided by the pilot—is rather robust to external perturbing forces of up to 100 N, which are significantly higher than potential flight perturbations originated by wind gusts or irregularities in the surface contact. Besides, extensive validation experiments (>200 flights) in different conditions were performed in an outdoor testing scenario and in a real refinery. All these experiments were fully successful and validated the proposed robot and its operating procedure as useful tools for contact inspection in real settings. Certified inspectors together with certified pilots confirmed the suitability of the proposed inspection solution during the refinery experiments.

The extension of the developed aerial robot to other industries using other sensors in the end-effector and the development and integration of end-user functionalities to autonomously implement B-scan and C-scan inspections using the automatic vision-based relative localization assistant described in Section 5.3 are the object of current development.

## 8. Patents

The mechanical configuration of the aircraft was patented in ES-2-614-994-B1 with the title “Aeronave con dispositivo de contacto” (in English: Aircraft with contact device) and was obtained the 23rd of February of 2018. The authors are, in order: Trujillo Soto, Miguel Ángel; Viguria Jiménez, Antidio; Márquez Font, José Carlos; Petrus Moreno, Ángel Luis; Jiménez Bellido, Antonio; García Freire, Juan Jesús and Ollero Baturone, Aníbal.

## Abbreviations

The following abbreviations are used in this manuscript:

COTS	Commercial off-the-shelf
DCM	Direct cosine matrix
DoF	Degree of freedom
EU	European Union
GNSS	Global navigation satellite system
GPS	Global positioning system
IMU	Inertial measurement unit
KW	Kilowatt
NDT	Non-destructive testing
RANSAC	Random sample consensus
RMS	Root mean square
ROI	Region of interest
RTK	Real-time kinematic
SURF	Speeded-up robust features
UAV	Unmanned aerial vehicle
UT	Ultrasonic testing
